# “You have to wait for a diagnosis first”: Barriers to preventive mental health support and early interventions for children and young people in Sweden

**DOI:** 10.1371/journal.pone.0351766

**Published:** 2026-06-16

**Authors:** Karin Törnbom, Dominique Hange, Eva-Lisa Petersson, Irene Svenningsson

**Affiliations:** 1 Primary Health Care/School of Public Health and Community Medicine, Institute of Medicine, The Sahlgrenska Academy, University of Gothenburg, Gothenburg, Sweden; 2 Department of Social Work, University of Gothenburg, Gothenburg, Sweden; 3 University of Gothenburg, Sahlgrenska Academy, Institute of Neuroscience and Physiology, Sahlgrenska Academy, University of Gothenburg, Gothenburg, Sweden; 4 Region Västra Götaland, Research, Education, Development & Innovation Primary Health Care, Gothenburg, Sweden; 5 Region Västra Götaland, Research, Education, Development & Innovation Primary Health Care, Research, Education, Development & Innovation Center Fyrbodal, Vänersborg, Sweden; Lahore College for Women University, PAKISTAN

## Abstract

**Background:**

Despite increasing attention to youth mental health, children and adolescents in Sweden experience fragmented, inequitable care with regional variation. Delays in diagnosis, limited preventive interventions, and poor inter-sectoral collaboration contribute to significant unmet needs. This study investigates system-level challenges and stakeholder perspectives on opportunities to enhance care pathways.

**Methods:**

We conducted a qualitative study in the Västra Götaland region, Sweden. Fourteen purposively selected participants – including senior executives, healthcare professionals, and parents took part in semi-structured interviews. We used systematic text condensation, according to Malterud, and the four steps involved in this method for analysing the interviews.

**Results:**

A central theme across interviews was the requirement for a formal diagnosis before children can access mental health support, particularly in school and primary care settings. Participants described this as a major barrier that delays early intervention and leaves children and young people with complex or atypical presentations without adequate support. Primary care professionals reported increasing mental health caseloads without corresponding increases in staffing or funding, limiting preventive work. Child and adolescent psychiatry (BUP) was described as overwhelmed, with long waiting times and limited continuity of care. A care manager within primary care was proposed as a way to help families navigate fragmented services and improve collaboration, although participants emphasised that such a role would need to be part of broader structural reform.

**Conclusions:**

Our findings highlight persistent systemic issues in mental health care for children and young people, including inequitable access, insufficient prevention, and fragmented collaboration across sectors. Strengthening primary prevention, reallocating resources to primary and school-based mental health care and implementing well-defined care coordination roles within broader restructuring may improve continuity and equity in service delivery. Comprehensive policy reform is needed to support person-centred, integrated care pathways for children and young people with mental health needs.

## Introduction

Mental health and illnesses are leading contributors to the global health burden, affecting between 10% and 20% of children and young people worldwide [1,2]. In most countries, the magnitude of mental health problems in young people has not been sufficiently acknowledged by governments and decision makers [3,4]. However, poor mental health causes disability in children and young people and has long-lasting effects throughout life [1].

In the Western world, care for children and young people with poor mental health faces significant challenges due to systemic deficiencies, both in paediatric specialist services and in primary care settings [5,6]. These issues are rooted primarily in inadequate service provision, insufficient training for healthcare professionals, and a lack of integrated care models [7]. For example, child and youth mental health may have been viewed as less of a problem, even in resource-rich countries [4]. Furthermore, mental health and poor health among youth have increasingly been addressed through secondary prevention, despite evidence supporting the potential of primary preventive strategies to promote mental health in young people [8].

Preventing common mental disorders (CMDs) in children and young people is crucial. It can maintain long-term mental and cognitive health, resilience, and social development, reducing the risk of chronic issues later in life [9–11]. Preventive measures are also critically important to ensure that the child is able to complete their education, as a positive school experience lays the foundation for life. Without qualifying grades, entering the labour market becomes significantly more difficult [12]. Preventive programmes should focus not only on children and young people but also on their families, aiming to strengthen resilience and reduce risk factors [10,13]. Mental health care for children and young people is delivered through a complex system involving multiple stakeholders, with regional variations in its structure and organisation [14,15]. Therefore, the system is often experienced as difficult to navigate, follow, and understand, especially when timely access to appropriate care is critical for children and young people seeking help [16,17]. In addition, care becomes fragmented, as collaboration can be hindered by differing guidelines, legal frameworks, funding structures, and organisational conditions. Similar challenges have been reported both in Sweden and internationally, highlighting the widespread nature of fragmented service delivery and limited inter-professional collaboration [18].

A significant lack of coordination and collaboration has been identified, particularly between primary care, specialist care, and schools [19]. Moreover, previous research has shown that addressing poor mental health in children and young people requires a cross-sectoral approach that seeks shared solutions [20–22]. Tackling these issues demands that stakeholders develop shared understandings and a collective commitment to joint action [22]. For such efforts to succeed, collaboration must be multifaceted, adaptable, and function effectively across multiple levels [20,23].

One of the most critical challenges to successful collaboration has been establishing a shared sense of commitment and clear collective responsibility among the partner organisations [24,25]. Therefore, to improve collaboration, it is necessary to explore the perceived challenges among various stakeholders and their managers, not only within healthcare but also across social services, schools, families, and student health services [24]. Such knowledge can guide the development of targeted improvement efforts aimed at reducing fragmentation and enhancing care and support for children, young people and their families.

Although prevention strategies are essential, the capacity of the healthcare and welfare system to coordinate and deliver timely support remains a major structural challenge. Fragmentation, unclear responsibilities, and limited cross-sectoral collaboration may undermine both preventive and treatment efforts. Therefore, understanding how key stakeholders perceive these systemic challenges is vital. To our knowledge, no studies have explored the perceived challenges of delivering care and support for children and young people with poor mental health from the perspective of involved managers and essential stakeholders.

The aim was to explore the experiences and perceptions of senior executives, professionals, and parents regarding the healthcare system’s ability to support children and young people with poor mental health and their families, and to identify improvements.

## Materials and methods

### Study design

This study was conducted in the Vastra Gotaland region, a large area located in southwestern Sweden.

The current study is part of a broader, comprehensive initiative aimed at improving care pathways and collaboration in the management of mental poor health among children and young people. Previous studies within this initiative have focused more specifically on how care pathways function within primary care for adults with poor mental health. How care and collaboration around this patient group can be improved within primary care has been examined through the care model “Co Work Care,” where care managers and rehabilitation coordinators work proactively to facilitate return-to-work through person-centred dialogue meetings with patients’ employers [26–30]. Our research group has subsequently identified a need to examine the care process for young people with poor mental health more specifically, as well as how a care manager can function for children and young people. With the current study, we broadened the perspective to focus on all major entities involved in the care of children and young people with poor mental health, according to the following design.

A qualitative study design was employed to investigate participants’ thoughts and experiences, emphasising the generation of professional insights and contextual understanding [31,32]. The study adheres to the Standards for Reporting Qualitative Research (SRQR) guidelines [33]. All the collected material was used for the current study only.

Authors conducting this research consisted of: KT, a female PhD and SWR with extensive knowledge in the field of qualitative research and experience of working with young people with poor mental health, the second author (DH) is a female associate professor and physician with extensive clinical and research experience in the field of mental health and in qualitative research, the third author (E-LP) is a female associate professor and OT with an extensive experience in qualitative research with limited experience working with children and the last author (IS) is a female associate professor and RN with extensive experience in qualitative research as well as professional experience working with children in health services and with children and their parents in primary healthcare settings.

### Study participants

The participants were purposefully selected to ensure a comprehensive exploration of the research question from various perspectives [32]. To achieve both a broad and in-depth understanding of the research question [34], senior executives within the organisations were selected together with experienced professionals working closely with children and young people experiencing poor mental health and their families. To ensure that the discussions and knowledge generated were relevant and tailored to the needs of children and young people, parents of young people with poor mental health were included to share their experiences with mental health services. Two parents also participated as patient and public involvement (PPI) representatives to validate the results from their perspective as stakeholders [35].

All the participants had extensive experience and a long work history of at least ten years, working with children and young people in the field of mental health. The participants were selected by the research team and their colleagues, who approached experienced individuals within the organisations. Recruitment focused on professionals with extensive experience of the child and youth mental health system. All individuals who were invited to participate agreed to take part in the study, and participation was entirely voluntary. None of the authors had any prior professional or personal relationships with the participants before recruitment.

Subsequently, the study consisted of 14 individuals from various organisations involved in the care of children and young people with poor mental health. Participants were purposively selected to represent a range of services responsible for children and young people’s mental health, in order to capture a broad diversity of experiences and perspectives. The sample size was considered sufficient as participants represented key roles across relevant services and had extensive professional experience. During the analysis, recurring patterns and themes were identified across interviews, and additional interviews were judged unlikely to generate substantially new insights, suggesting that data saturation had been reached. Sample size was according to Malterud further guided by the concept of information power, whereby the adequacy of the sample was continuously assessed in relation to the study aim, recognising that samples holding rich and relevant information require fewer participants [36].

The fact that all who were asked agreed to participate was interpreted as an indication that the questions under study were relevant and important to them. All participants signed a written informed consent form, which was then returned to the research group via email or postal mail. To protect the participants’ anonymity, their professional positions and education levels are presented separately as follows:

The study included managers in primary care, specialised care for children, unit managers for a clinic that offers specialist support for children and youth with poor mental health, and managers at the youth clinic. There were also representatives from four different primary care centres, a school and two paediatric services.

Educational background: Paediatric nurses working in paediatric medicine (n = 2), physicians specialising in paediatric and adolescent medicine (n = 2), general practitioners (n = 3), psychologists and licenced psychotherapists (n = 1), specialist nurses and care managers (n = 2), social workers (n = 1), midwives (n = 1), and parents of children with relevant healthcare experience (n = 2).

### Human ethics and consent to participate

All participants signed a written informed consent form, which was then returned to the research group via email or postal mail.

### Data collection

All the materials were collected through individual interviews conducted via teams, which facilitated scheduling for employees with various fields of responsibilities and a high workload. It was also advantageous considering the long travel times between different workplaces and likely helped reduce the risk of potential dropouts. Each interview was audio-recorded and subsequently transcribed verbatim by the research team. Data collection took place between July 8th and September 4th of 2024. Interviews lasted between 30 minutes and one hour and were conducted by the authors (KT), (E-LP), and (IS), none of whom had prior relationships with the participants.

The interview guide was developed specifically for this study and was informed by the researchers’ professional and research experience in the field. Two members of the research team with extensive experience in the field were consulted during the development of the interview guide to help identify relevant questions for the participants. The interview guide covered three main areas: experiences of the healthcare system for children and young people with poor mental health, perspectives on early intervention and preventive work, and collaboration between services involved in the care of children and young people. Examples of questions covered in the interviews included: *How do you perceive the healthcare system functioning for children and young people with mental ill health and their families? How do you view the possibilities for preventive work in children’s mental health?* And *how does collaboration function between the different services involved in supporting children and young people with mental ill health?*

The participants discussed their experiences of how the care process works for children and young people with poor mental health, as well as how the collaboration around this target group is structured and functions. The interview guide was semi-structured, and follow-up questions were adjusted according to the direction chosen by the participants. The interviewers (KT), (E-LP) and (IS) encouraged participants to speak freely within the frame of the subject, encouraging more personal reasoning and reflections to emerge. In most cases, participants chose to discuss the healthcare system and the collaboration surrounding children and young people in these systems.

### Analysis

The data were analysed via Systematic Text Condensation (STC) as outlined by Malterud [36]. This qualitative analysis method was selected because of its pragmatic, holistic, exploratory, and reflexive approach, which aligns with our overarching goal of presenting a nuanced understanding of the participants’ experiences. The analysis was based on transcriptions of the interviews. To ensure participant anonymity, all the participants were pseudonymized. The first author (KT) took primary responsibility for the analysis, with input, comments, and feedback from the other authors (IS), (E-LP) and (DH). In the first step, to establish an overview of the data, 1) the authors individually read the interviews in their entirety to create a broader analytic space. As the interviews were conducted in Swedish and later translated into English, the authors collaborated during the analysis to ensure accurate interpretation of the data. 2) Various aspects of the participants’ experiences with the care process for children and youth with mental illness and the professional collaboration surrounding this work were identified and labelled as different meaning units. 3) In the third step, the codes were independently abstracted, progressing from code to meaning. Codes that were related were grouped, with these groups being progressively condensed and refined through discussions among all the authors. During the formation of the condensed code groups, the original terminology used by the participants was preserved to ensure that the perspectives of all participants who provided information in this specific area were adequately represented. 4) Finally, the contents of the condensed code groups were synthesised. This step primarily involved ensuring that a reliable narrative was produced, one that satisfactorily addressed the study’s research questions.

Any analytical disagreements were discussed among the authors until consensus was reached. To enhance the rigour and trustworthiness of the analysis, the process involved continuous dialogue among the authors, iterative review of the coding and categorisation, and careful documentation of decisions throughout the process.

## Results

The analysis process *resulted in the following overarching codes: 1) The healthcare system fails children and young people with poor mental health and their families; 2) Early intervention and mental health prevention – the central role of primary care; 3) Breaking silos – challenges and solutions for better collaboration;* 4) *The school health service sees all children and is therefore an important partner to collaborate with;* 5) *Addressing the challenges and needs in child and youth psychiatry;* and 6) *Being a coordinator for children and young people with poor mental health as a part of the solution.*

### The healthcare system fails children and young people with poor mental health and their families

Participants described the healthcare system for children and young people with poor mental health as difficult for families to understand and navigate. Support differed between areas within the region. For example, parent-supportive counselling was offered at some primary care centres, while similar support was classified as psychiatric care elsewhere. Participants perceived these differences as confusing for families and as contributing to unequal access to care depending on geographical location.

*Sometimes the young person might say, ‘However, my friend in S-town got CBT sessions for their health anxiety at the health centre, so why can’t I get that here in M-town?’ And I can’t truly answer that - it’s just how things are today. Different things are available at different PCCs (GP, Marie)*

The participants argued that the systems are not designed with the individual’s best interests in mind, a shortcoming that becomes particularly evident for caregivers of children with complex needs. These systems are especially challenging to navigate for children with multiple diagnoses and their families, often requiring considerable time before appropriate support is identified and accessed.

*You’d think that the kids with the greatest needs would get the most help, but in my experience, it’s actually the opposite. If you don’t fit into the right track—like the ‘ADHD’ track or the ‘depression’ track—then you’re often seen as too complicated… You don’t fit anywhere. No one thinks they have the right expertise to take you on (Specialist nurse, Ingegerd)*

The participants also emphasised the need for more leisure activities tailored to children and young people with various diagnoses. Several studies have noted that children with conditions such as autism or ADHD are at a greater risk of problematic school absenteeism or becoming socially isolated in front of screens due to a lack of adequately adapted programmes and activities.

*If you’ve autism, ADHD or some mild cognitive disability, you might not fit into a regular kids’ group for activities such as horseback riding or soccer. Then, you end up excluded there, just like you are at school, and the risk of that child becoming even more isolated increases… That’s why I’m saying, let’s add more preventive options here too! Like horseback riding for autistic kids, or similar. (Parent, Christina)*

Participants identified another group of children and young people who were described as falling between services. These were children who did not improve through the limited interventions available in primary care but were not considered ill enough for specialist child and adolescent psychiatry (BUP). Participants described them as stranded in a “no-man’s land”, with few available options. In some areas, Youth Mental Health Services (Ungas Psykiska Hälsa, UPH) offered group activities or individual counselling, whereas in other areas such services were underdeveloped or unavailable.

### Early intervention and mental health prevention – the central role of primary care

Participants called for earlier intervention, before problems escalated. They described the current system as too reactive and argued that waiting times could contribute to “unnecessary mental ill health”. Delays were also perceived as affecting both wellbeing and school performance. The requirement for a child to have a formal diagnosis to qualify for support was identified as a significant and limiting flaw within the system:

*This means that the child must wait, sometimes a very long time, just to obtain a diagnosis before they can obtain the right help at school. It would be better if we could start by asking, “What difficulties does the child actually have?” and then build support around that. In this way, help is based on what the child needs, not on a diagnosis. For a child, waiting years for a diagnosis feels like forever—and in that time, they can miss out on many important developments (Psychologists and licenced psychotherapists, Britta)*

At the same time, it was argued that both mental health expertise and resources need to be strengthened within primary care. Primary care managers argued that if primary care is expected to support children whose difficulties are not classified as psychiatric disorders, additional resources are needed. Several participants perceived that the workload related to children and young people’s mental health had increased without a corresponding increase in staffing or funding.

*We are currently handling increasingly complex cases, such as neuropsychiatric disabilities, PTSD, and orthorexia. We’re taking on a lot from specialist healthcare. However, have I received more funding? No. Have I gotten more staff? My answer is no. (Physician and paediatric specialist, Sonja)*

All the participants expressed a desire to focus more on primary prevention but also noted that, unfortunately, this approach was not prioritised within their organisations. Leaders at the primary care level explained that they receive compensation for diagnosing conditions and managing severe issues but not for implementing preventive interventions, such as youth discussion groups or parent counselling. These leaders viewed this as an incorrect approach to care and were strongly convinced that preventive work needs to be given significantly greater emphasis and support.

*We need to be able to perform more primary prevention. Right now, we’re almost exclusively working on secondary prevention at the PCCs, and most of the people who come to us are already sick. Unfortunately, we cannot focus on prevention because the funding has to go to those who are already ill. Even though the greatest benefit lies in working preventively. If you can support the whole family early on, before you have to wait a year for BUP and potentially become very ill, you can do an incredible amount to change the course of things. (GP and paediatric specialist, Margit)*

At some PCCs, Youth Mental Health Services (UHPs) were available and functioned well as supportive resources. However, in other areas, UPHs are either absent or overburdened, resulting in long waiting lists. The participants therefore noted that while the help provided by the UPH was very effective and highly valuable in complementing child and adolescent psychiatric services (BUP), it also contributed to unequal access to care.

### Breaking silos – challenges and solutions for better collaboration

The participants emphasised the importance of improving communication among key stakeholders. The consensus was that if you are currently responsible for an issue, you should take ownership and address it directly with the relevant party. Many problems can be resolved if individuals take responsibility for their own tasks rather than shifting the burden to other departments. Another challenge identified was the use of different “languages” across various services, which can lead to a lack of trust between them. A lack of understanding of each other’s roles can also create unrealistic expectations, such as the belief that “if the child just gets to BUP, everything will be fine,” or that “student health services can fix everything.”

*Specialist healthcare workers need to talk more about habilitation, primary care, and schools. We all need to sit down and discuss our own areas of responsibility so that we do the right thing for the child. Just like we do in SIP meetings, where we truly take joint responsibility for the children and their families. However, this does not happen enough today; some call it silos, while others call it ‘stovepipes. (Social worker, Lisa)*

Many participants stated that collaboration issues are not new but have been discussed for several decades. The general consensus was that the problems with collaboration are inherent in the structure itself.

*When I was cleaning out the office, I was shocked to see that I could find the exact same collaboration issues in binders from the 1980s and 1990s. It says: ‘We need to talk more to each other (professionals from different fields),’ ‘We need to trust each other and listen to each other.’ And yet, we still haven’t managed to fix this. We still have a healthcare system where there is no collaboration. (Psychologist and licenced psychotherapist, Alex)*

Some participants described the reimbursement system as a barrier to collaboration. Since revenue was linked to patient visits, time spent on interprofessional coordination was not always supported financially. Under conditions of limited resources and high workload, staff tended to focus on narrowly defined tasks. Collaboration was therefore perceived as time-consuming and difficult to prioritise. To promote collaboration, the participants agreed that its benefits should be clearly communicated by leadership and that new employees, in particular, need clear guidance on why collaboration is essential and how it can be put into practice.

There was consensus on the importance of creating more sustainable and lasting systems for collaboration. Notably, most people today struggle to establish effective collaboration, despite everyone recognising its need. However, owing to the lack of simple systems of cooperation, staff often attempt to find their own solutions, and if these prove difficult, it is easy to give up.

The participants also agreed that problems with collaboration lead to poorer care and can result in long-term, often unnecessary issues for children and young people.

*A child with speech and attention issues, identified as early as age 3, might wait up to 2.5 years before receiving help. That is an incredibly long wait for a preschooler. However, the complicated referral process makes everything so slow—first to a paediatrician, then to a psychologist, a speech therapist, and then to contact preschool. If these steps were coordinated simultaneously, the wait time could be reduced to just a few months, an invaluable difference for the small child. (GP, Ann-Sofie)*

### The school health service sees all children and is therefore an important partner to collaborate with

The participants emphasised that student health services are important because they provide access to all children, allowing for the early identification of issues. Some have argued that student health services should receive increased resources to enable clearer assessments and facilitate closer collaboration with primary care providers.

*A well-functioning school health service is essential; they have contact with all the children, and they see everyone. Therefore, if they can identify issues and write reports for us in primary care, we know more when we take over the children. Right now, it seems there’s a lack of resources in that area. (Specialist nurse and care manager, Anna)*

The participants noted, however, that in many areas, school health services were severely understaffed, making it very difficult to find someone who was familiar with and could speak to a specific case. The level of support also varies across different municipalities. In some areas, a well-developed school health service was available, featuring psychologists, social workers, educators, and nurses who collaborated effectively with primary care and social services.

### Addressing the challenges and needs in child and youth psychiatry

The participants described a child and youth psychiatry system that is currently struggling to cope. There was a unanimous perception that service has rapidly deteriorated in recent years. Leaders at all levels recognised this as an escalating issue characterised by long waiting times and a high rate of returned referrals.

*BUP sends back many referrals that need to be supplemented in various ways… I don’t know if they’re doing it just to buy time? Then, they write to us saying, ‘Unfortunately, we cannot provide safe patient care at the moment.’ Therefore, they’re very clear and open about the fact that they lack the necessary resources. (GP, Malin)*

The participants described how children often have to wait for many months, even up to years, to be seen by child and adolescent psychiatry (BUP). During this waiting period, many children’s mental health worsened, and at the same time, it was noted that both the children and their parents often placed high expectations on the help they would eventually receive from BUP.

*Many parents think, ‘If we just get to BUP, everything will be fine,’ but then they might only get a handful of visits there and... what happens after that? Many are considered too healthy or not truly suited for BUP, and then they might not receive any more help or have to return to the waiting list. (Physician and paediatric specialist, Sonja)*

In primary care, it is often noted that referring children or young people to BUP can be disheartening. There is already an expectation that it will take a long time if the family even receives help at all. The prevailing sentiment was that the issue of mental health problems among children and young people seems endless and overwhelming. The system intended to relieve pressure on BUP, “One Pathway In,” was seen as something too early to evaluate. Several managers described that it had not yet functioned as originally intended, but perhaps it needed more time to do so.

The participants also agreed that there should not be an end goal of simply “saving BUP” and that directing many resources toward BUP was not the right approach. Instead, they believed that children and young people are most in need of simpler, quicker interventions rather than additional psychiatric resources. Moreover, they acknowledged that BUP requires more trained specialists and an increase in the number of care facilities. This would help retain staff within the BUP and reduce the waiting times for an already overburdened service.

The participants agreed that the starting point should be the question, “What do children and young people truly need?” and that the system should be built around those needs.

### Being a coordinator for children and young people with poor mental health as a part of the solution

The participants agreed that care for children and young people with poor mental health needs to be significantly improved. In addition to recognising a growing need for goal-oriented and well-initiated collaboration, there was a desire for more accessible and preventive interventions to offer children and their families.

Participants were uncertain and divided regarding the need for a care coordinator within primary care. Their hesitation was mainly linked to the scarcity of resources. They argued that any new role would need to be carefully considered and evaluated in relation to other possible uses of the same resources. Some participants believed that a care coordinator is needed precisely because the system is so complicated. In such a system, children and young people need guidance and support. Who should they turn to? Is there someone who will manage their contacts and care process? In these matters, a care coordinator was potentially very helpful. There was a demand for a care coordinator who could highlight the need for collaboration and possibly even oversee and guide this collaboration. However, the participants did not want to shift the responsibility for collaboration to an additional resource that might not always be available.

The participants also wanted a person with evident expertise, ideally with extensive experience in the field of young people’s poor mental health.

*This needs to be a truly competent person if it is going to add any value. You need to understand how the municipality and region work and what help is available to be able to guide people correctly. In addition, you need to have a lot of expertise in the area to help the staff work together in the best way... yeah, good luck with that, I’d say. It is not easy to create a resource that actually adds value in this complicated system. (Psychologist and licenced psychotherapist, Britta)*

The participants’ greatest wish was for the system and support for children and young people to be completely restructured and expanded. They expressed a desire for a competent steering group to review the entire system and make healthcare more accessible and tailored to the right needs. The participants believed that a care coordinator could be a part of such a new system, but they did not think that this new resource alone could solve the extensive shortcomings currently present in the healthcare system.

## Discussion

The findings reveal a fragmented and regionally inconsistent healthcare system that limits equitable access to mental health support for children and young people. Children and young people with complex or multiple needs are particularly disadvantaged, and a substantial group fall between primary care and specialist services without receiving adequate support.

Preventive and early interventions were not sufficiently prioritised, with long waiting times and diagnosis-based eligibility contributing to worsening mental health. Primary care faces increasing demands without proportional resources, while specialist child and adolescent psychiatry is perceived as overloaded.

The participants in the present article discussed systemic shortcomings within the healthcare system for children and young people with poor mental health and their families. They described a system that is fragmented and inequitable, aligning with prior international qualitative findings that report similar deficiencies in clear communication and consistent pathways for care [37,38]. According to the participants, care offerings vary significantly between regions, complicating families’ efforts to seek support and imposing differential access based on geography. Comparable challenges were also identified in reviews of national mental health policies, where inconsistent approaches have perpetuated service gaps [39–41].

The inconsistency in service availability extends to preventive and early intervention strategies. Evidence suggests that children and young people with complex needs are particularly disadvantaged, facing delays due to the requirement for formal diagnoses before intervention can be initiated [42]. This waiting period exacerbates developmental and academic setbacks for children and becomes extremely frustrating for families [43,44]. Furthermore, research has emphasised that a reactive rather than proactive approach leads to overwhelming workloads for health care professionals, primarily due to more youth with mental ill health but no increase in resources [6,37]. In addition, with respect to classification, labelling problems under specific diagnostic categories such as ADHD or depression not only takes a long time to investigate but also leaves children with multiple or atypical diagnoses in limbo, delaying crucial support [6,37,45].

The participants highlighted that the existing compensation system reinforces a focus on secondary prevention, treating poor mental health after they manifest, instead of emphasising early or primary prevention. There is a great need to shift the emphasis toward primary prevention to address children and young people’s mental health needs at an early stage, when they first arise [8]. In Swedish regions where Youth Mental Health Services are available, their effectiveness is acknowledged; however, the uneven distribution of these services exacerbates existing inequities [46]. This observation is consistent with reviews highlighting the need for a more equitable distribution of mental health resources to prevent unnecessary delays in treatment [47].

Limited collaboration between services emerged as another important barrier. Participants described persistent siloed practices, despite longstanding calls in Sweden for stronger interdisciplinary collaboration around children and young people with poor mental health. This finding is consistent with previous research showing that collaboration is often difficult to sustain when financial and administrative systems prioritise productivity over continuity and integrated care [37,38]. Primary care providers are also often fatigued by the multitude of guidelines they are expected to follow, guidelines that are not always designed in alignment with the realities of primary care practice or its practical working conditions [48,49].

Student health services were described as particularly important because they have regular contact with almost all children and young people. When adequately resourced, these services may help identify early signs of mental health difficulties and provide information that supports assessments in primary care [37,50]. This multidisciplinary approach was shown to improve care continuity and prompt referrals to specialised services, fostering collaboration between schools and healthcare [51,52].

In addition to the strengths and limitations in school health services, BUP is under significant strain [40]. The participants described BUP as handling overwhelming caseloads with long waiting periods that exacerbate children’s conditions; delays in treatment during these waiting periods contribute not only to further deterioration in mental health but also to increasing frustration and disillusion among families [40,52]. Several studies have indicated that rapid referral and swift intervention models can ease these effects [53,54]. The expectation that the BUP will provide a definitive solution is often unmet. Once children are referred, the limited number of consultations frequently results in waiting lists or ambiguous follow-up strategies. This reflects a broader deficit in resource allocation and a system that remains oriented mainly towards reactive rather than preventive care [55,56].

The participants in our study discussed the role of a care coordinator within primary care as a potential strategy to navigate an overly complex mental health system. Proponents of this model argue that a dedicated care manager could streamline communication, facilitate interdisciplinary teamwork, and ensure that families receive continuous, holistic care across various services [37,51,57]. However, the introduction of such a role raises concerns about resource allocation: any new funding directed toward a care manager could detract from other critical services if not implemented within a broader structural reform [53,55]. As one participant noted, the value of a care coordinator is contingent on its ability to navigate complex interagency relationships and contribute meaningfully to collaborative care practices. This task requires both experience and sustained intersectoral cooperation [55,58]. Furthermore, evidence from collaborative care models suggested that coordination roles are most effective when embedded within formal operational frameworks rather than introduced as informal additions to existing services [59].

### Strengths and limitations

A major strength of this study is the inclusion of all key stakeholder involved in the delivery and receipt of care and support for children and youth with poor mental health, including managers at all levels of the care process, as well as families. Despite representing different disciplines, the participants were surprisingly consistent in their reflections and experiences. They also expressed strong self-criticism towards their own organisations, which demonstrated a notable openness and honesty during the interviews. Their extensive experience working with children and young people with mental health problems at various levels of care provided rich, well-informed perspectives on the topic. A limitation of this study is the relatively small sample and the predominance of professionals and senior stakeholders among the participants. While this focus provided insight into organisational structures and system-level challenges, the limited inclusion of service users means that the findings may not fully reflect the experiences of children and families navigating the system. However, the emphasis on professionals and decision-makers was consistent with the study’s aim of exploring structural conditions and collaboration within youth mental health services.

The management perspective may also differ from the experiences of frontline staff or service users, which could affect the breadth of insight into everyday clinical practice. The results are also context-specific, reflecting the organisational and geographical setting of the study, and may not be directly applicable to other contexts. Another limitation is that the study relies on participants’ perceptions of increasing demand, long waiting times, and system strain. Consequently, the findings should be understood as reflecting experienced system pressures rather than objectively measured service trends. As in all qualitative research, the interpretation of findings is shaped by the researchers’ preunderstandings and analytical decisions, which we sought to mitigate through transparency and reflexivity in reporting.

## Conclusion

The findings from this qualitative study suggest that participants experienced the healthcare system for children’s mental health in western Sweden as fragmented and regionally inconsistent, with perceived barriers related to diagnostic thresholds, limited preventive support, and challenges in collaboration between services. Children and young people with complex or overlapping needs were described as particularly vulnerable to falling between organisational boundaries.

Given the regional nature of the study, the findings should be understood as reflecting stakeholder experiences and perceptions rather than providing generalisable evidence of system-wide effects. Nevertheless, the results point to important areas for further development and evaluation. These include strengthening early and preventive support, enhancing the capacity of primary care, improving collaboration between schools, primary care and specialist services, and considering coordinated support functions for families navigating the care system.

## References

[1] Kieling C, Baker-Henningham H, Belfer M, Conti G, Ertem I, Omigbodun O, et al. Child and adolescent mental health worldwide: Evidence for action. Lancet. 2011;378(9801):1515–25. doi: 10.1016/S0140-6736(11)60827-1 22008427

[2] Serazzi F, Barbic F, Stranges S. Public health prevention programmes for mental illness in school-age children and adolescents. Eur J Public Health. 2024;34(Suppl 3):ckae144.2257. doi: 10.1093/eurpub/ckae144.2257

[3] Remschmidt H, Belfer M. Mental health care for children and adolescents worldwide: A review. World Psychiatry. 2005;4(3):147–53. 16633533 PMC1414760

[4] Belfer ML. Child and adolescent mental disorders: The magnitude of the problem across the globe. J Child Psychol Psychiatry. 2008;49(3):226–36. doi: 10.1111/j.1469-7610.2007.01855.x 18221350

[5] Fatima S, Brenisin K, Doyle I, Gathii E, Breen K. Collaborative care for mental health of children and young people: A qualitative study of the experiences of mental health professionals. IJHG. 2024;29(2):149–61. doi: 10.1108/ijhg-12-2023-0124

[6] Carbonell Á, Georgieva S, Navarro-Pérez J-J, Prades-Caballero V. The hodgepodge reality: A qualitative systematic review of the challenges and barriers in child and adolescent mental health care systems. Adolescent Res Rev. 2023;9(3):563–86. doi: 10.1007/s40894-023-00227-7

[7] Limb M. Children are being failed by substandard mental health services. BMJ. 2017;358:j3641. doi: 10.1136/bmj.j3641 28751546

[8] Colizzi M, Lasalvia A, Ruggeri M. Prevention and early intervention in youth mental health: Is it time for a multidisciplinary and trans-diagnostic model for care?. Int J Ment Health Syst. 2020;14:23. doi: 10.1186/s13033-020-00356-9 32226481 PMC7092613

[9] Ormel J, VonKorff M. What should a nation do to prevent common mental disorders? Meet seven conditions for effective prevention. Mental Health & Prevention. 2024;33:200320. doi: 10.1016/j.mhp.2024.200320

[10] McGovern R, Balogun-Katung A, Artis B, Bareham B, Spencer L, Alderson H, et al. The effectiveness of preventative interventions to reduce mental health problems in at-risk children and young people: A systematic review of reviews. J Prev (2022). 2024;45(4):651–84. doi: 10.1007/s10935-024-00785-z 38884876 PMC11271346

[11] Serazzi F, Barbic F, Stranges S. Public health prevention programmes for mental illness in school-age children and adolescents. European Journal of Public Health. 2024;34(Supplement_3). doi: 10.1093/eurpub/ckae144.2257

[12] Rahmani H, Groot W. Risk Factors of Being a Youth Not in Education, Employment or Training (NEET): A scoping review. International Journal of Educational Research. 2023;120:102198. doi: 10.1016/j.ijer.2023.102198

[13] Kuhn ES, Laird RD. Family support programs and adolescent mental health: Review of evidence. Adolesc Health Med Ther. 2014;5:127–42. doi: 10.2147/AHMT.S48057 25177156 PMC4096456

[14] Grummitt L, Barrett E, Halladay J, Bailey S, Birrell L, Hunter E, et al. Embedding action on social and structural determinants of mental health into a national framework: An “immunisation schedule” for the prevention of common mental disorders. Mental Health & Prevention. 2023;32:200308. doi: 10.1016/j.mhp.2023.200308

[15] Arruda W, Bélanger SA, Cohen JS, Hrycko S, Kawamura A, Lane M, et al. Promoting optimal mental health outcomes for children and youth. Paediatr Child Health. 2023;28(7):417–36. doi: 10.1093/pch/pxad032 37885601 PMC10599492

[16] Helander M, Fredriksson M, Lohela-Karlsson M. Exploring stakeholders’ perceived problems associated with the care and support of children and youth with mental ill health in Sweden: A qualitative study. J Health Popul Nutr. 2024;43(1):30. doi: 10.1186/s41043-024-00520-8 38378621 PMC10880220

[17] Richter Sundberg L, Gotfredsen A, Christianson M, Wiklund M, Hurtig A-K, Goicolea I. Exploring cross-boundary collaboration for youth mental health in Sweden - A qualitative study using the integrative framework for collaborative governance. BMC Health Serv Res. 2024;24(1):322. doi: 10.1186/s12913-024-10757-y 38468279 PMC10929090

[18] Mubarak N, Hatah E, Aris MAM, Shafie AA, Zin CS. Consensus among healthcare stakeholders on a collaborative medication therapy management model for chronic diseases in Malaysia; A Delphi study. PLoS One. 2019;14(5):e0216563. doi: 10.1371/journal.pone.0216563 31075110 PMC6510413

[19] O’Malley D, Woods-Jaeger BA, Dowd MD. Building a collaboration between a children’s hospital and an early childhood education and social services center. Curr Probl Pediatr Adolesc Health Care. 2017;47(9):222–8. doi: 10.1016/j.cppeds.2017.07.008 28826807

[20] Bryson JM, Crosby BC, Stone MM. Designing and Implementing Cross‐Sector Collaborations: NeededandChallenging. Public Administration Review. 2015;75(5):647–63. doi: 10.1111/puar.12432

[21] Fjellfeldt M. Developing long-term sustainable collaborations between welfare providers that support and promote child and youth mental health in Sweden-A qualitative interview study. Int J Environ Res Public Health. 2022;19(13):7730. doi: 10.3390/ijerph19137730 35805389 PMC9265848

[22] Stubbing J, Gibson K. Can We Build “Somewhere That You Want to Go”? Conducting Collaborative Mental Health Service Design with New Zealand’s Young People. Int J Environ Res Public Health. 2021;18(19):9983. doi: 10.3390/ijerph18199983 34639289 PMC8507894

[23] Anvik C, Waldahl RH. Sustainable collaboration to support vulnerable youth: mental health support teams in upper secondary school. SI. 2018;6(3):282–8. doi: 10.17645/si.v6i3.1525

[24] Widmark C, Sandahl C, Piuva K, Bergman D. Barriers to collaboration between health care, social services and schools. Int J Integr Care. 2011;11:e124. doi: 10.5334/ijic.653 22125502 PMC3225277

[25] Widmark C, Sandahl C, Piuva K, Bergman D. Parents’ experiences of collaboration between welfare professionals regarding children with anxiety or depression - An explorative study. Int J Integr Care. 2013;13:e045. doi: 10.5334/ijic.986 24198739 PMC3817954

[26] Björkelund C, Saxvik A, Svenningsson I, Petersson E-L, Wiegner L, Larsson M, et al. Rehabilitation cooperation and person-centred dialogue meeting for patients sick-listed for common mental disorders: 12 months follow-up of sick leave days, symptoms of depression, anxiety, stress and work ability - a pragmatic cluster randomised controlled trial from the CO-WORK-CARE project. BMJ Open. 2023;13(6):e074137. doi: 10.1136/bmjopen-2023-074137 37295824 PMC10277141

[27] Petersson E-L, Törnbom K, Björkelund C, Jerlock M, Hange D, Udo C, et al. Process evaluation of the CO-WORK-CARE model: Collaboration and a person-centred dialogue meeting for patients with common mental disorder in primary health care. Scand J Caring Sci. 2024;38(3):602–13. doi: 10.1111/scs.13268 38718100

[28] Petersson E-L, Törnbom K, Hange D, Nejati S, Jerlock M, Wikberg C, et al. The experiences of care managers and rehabilitation coordinators of a primary care intervention to promote return to work for patients with common mental disorders: A qualitative study. BMC Fam Pract. 2020;21(1):272. doi: 10.1186/s12875-020-01348-x 33339512 PMC7749497

[29] Saxvik A, Svenningsson I, Törnbom K, Petersson E-L, Björkelund C, Gabartaite G, et al. GPs’ experiences of a collaborative care model for patients with common mental disorders who need sick leave certification: A qualitative study. BJGP Open. 2022;6(4):BJGPO.2022.0042. doi: 10.3399/BJGPO.2022.0042 35977733 PMC9904781

[30] Saxvik A, Törnbom K, Petersson E-L, Hange D, Nejati S, Björkelund C, et al. Experiences of patients with common mental disorders concerning team-based primary care and a person-centered dialogue meeting: An intervention to promote return to work. PLoS One. 2022;17(7):e0271180. doi: 10.1371/journal.pone.0271180 35802679 PMC9269955

[31] Batalden M, Batalden P, Margolis P, Seid M, Armstrong G, Opipari-Arrigan L, et al. Coproduction of healthcare service. BMJ Qual Saf. 2016;25(7):509–17. doi: 10.1136/bmjqs-2015-004315 26376674 PMC4941163

[32] Dutton B, Humphrey N, Qualter P. Getting the pieces to fit: NHS and third sector collaboration to enhance crisis mental health service provision for young people. BMC Health Serv Res. 2023;23(1):307. doi: 10.1186/s12913-023-09198-w 36997929 PMC10061406

[33] O’Brien BC, Harris IB, Beckman TJ, Reed DA, Cook DA. Standards for reporting qualitative research: A synthesis of recommendations. Acad Med. 2014;89(9):1245–51. doi: 10.1097/ACM.0000000000000388 24979285

[34] Wan Z. Participant Selection and Access in Case Study Research. Challenges and Opportunities in Qualitative Research. Springer Singapore. 2019. 47–61. 10.1007/978-981-13-5811-1_5

[35] Totzeck C, van der Meer AS, Christiansen H, Durlach F, Li Sanchez K, Schneider S. Systematic review: Patient and public involvement of children and young people in mental health research. Clin Child Fam Psychol Rev. 2024;27(1):257–74. 38402358 10.1007/s10567-024-00470-x PMC10920437

[36] Malterud K. Systematic text condensation: A strategy for qualitative analysis. Scand J Public Health. 2012;40(8):795–805. doi: 10.1177/1403494812465030 23221918

[37] Darcy L, Råberus A, Sundler AJ. A qualitative analysis of child and family complaints related to child mental health services. J Child Adolesc Psychiatr Nurs. 2024;37(1):e12436. doi: 10.1111/jcap.12436 37443353

[38] Carbonell Á, Georgieva S, Navarro-Pérez J-J, Prades-Caballero V. The hodgepodge reality: A qualitative systematic review of the challenges and barriers in child and adolescent mental health care systems. Adolescent Res Rev. 2023;9(3):563–86. doi: 10.1007/s40894-023-00227-7

[39] Jonsson F, Goicolea I, Christianson M, Carson DB, Wiklund M. Landscapes of care and despair for rural youth - a qualitative study in the northern Swedish “periphery”. Int J Equity Health. 2020;19(1):171. doi: 10.1186/s12939-020-01288-z 33008434 PMC7531094

[40] Lourie IS, Hernandez M. A historical perspective on national child mental health policy. Journal of Emotional and Behavioral Disorders. 2003;11(1):5–9. doi: 10.1177/106342660301100102

[41] Jones EB, Lucienne TM. The integrated care for kids model: Addressing fragmented care for pediatric medicaid enrollees in seven communities. J Health Care Poor Underserved. 2023;34(1):503–9. doi: 10.1353/hpu.2023.0034 37464510

[42] Störbeck C. Early childhood development is not enough: In defense of children with developmental delays and disabilities and their right to family-centered early childhood intervention (in the global south). Children (Basel). 2024;11(5):606. doi: 10.3390/children11050606 38790601 PMC11119497

[43] Boulton KA, Hodge M-A, Jewell A, Ong N, Silove N, Guastella AJ. Diagnostic delay in children with neurodevelopmental conditions attending a publicly funded developmental assessment service: Findings from the Sydney Child Neurodevelopment Research Registry. BMJ Open. 2023;13(2):e069500. doi: 10.1136/bmjopen-2022-069500 36725093 PMC9896183

[44] Nowak HI, Broberg M, Starke M. Parents’ experience of support in Sweden: Its availability, accessibility, and quality. J Intellect Disabil. 2013;17(2):134–44. doi: 10.1177/1744629513486229 23644952

[45] Werkhoven S, Anderson JH, Robeyns IAM. Who benefits from diagnostic labels for developmental disorders?. Dev Med Child Neurol. 2022;64(8):944–9. 35191027 10.1111/dmcn.15177 PMC9306602

[46] Goicolea I, Hultstrand Ahlin C, Waenerlund A-K, Marchal B, Christianson M, Wiklund M, et al. Accessibility and factors associated with utilization of mental health services in youth health centers. A qualitative comparative analysis in northern Sweden. Int J Ment Health Syst. 2018;12:69. doi: 10.1186/s13033-018-0249-4 30459827 PMC6234690

[47] Marchionatti LE, Schäfer J, Karagiorga VE, Balikou P, Mitropoulou A, Serdari A. The mental health care system for children and adolescents in Greece: A scoping review and structure assessment. 2024. 10.3389/frhs.2024.1470053 PMC11668766 39723330

[48] Fernemark H, Karlsson N, Skagerström J, Seing I, Karlsson E, Brulin E, et al. Psychosocial work environment in Swedish primary healthcare: A cross-sectional survey of physicians’ job satisfaction, turnover intention, social support, leadership climate and change fatigue. Hum Resour Health. 2024;22(1):70. doi: 10.1186/s12960-024-00955-4 39443998 PMC11500482

[49] Samuelsson C, Gunnarsson L, Svärdman F, Rück C, Lindsäter E. Physicians’ experiences of assessing and supporting fatigued patients in primary care: a focus group study. BMC Prim Care. 2025;26(1):197. doi: 10.1186/s12875-025-02891-1 40490742 PMC12147317

[50] Love HE, Schlitt J, Soleimanpour S, Panchal N, Behr C. Twenty years of school-based health care growth and expansion. Health Aff (Millwood). 2019;38(5):755–64. doi: 10.1377/hlthaff.2018.05472 31059359

[51] Bartlett N, Freeze TB. Community Schools: New perspectives on the wraparound approach. Exceptionality Education International. 2018;28(2).

[52] Huang L, Stroul B, Friedman R, Mrazek P, Friesen B, Pires S, et al. Transforming mental health care for children and their families. Am Psychol. 2005;60(6):615–27. doi: 10.1037/0003-066X.60.6.615 16173894

[53] Acharya B, Maru D, Schwarz R, Citrin D, Tenpa J, Hirachan S, et al. Partnerships in mental healthcare service delivery in low-resource settings: developing an innovative network in rural Nepal. Global Health. 2017;13(1):2. doi: 10.1186/s12992-016-0226-0 28086925 PMC5237195

[54] Stephan SH, Weist M, Kataoka S, Adelsheim S, Mills C. Transformation of children’s mental health services: The role of school mental health. Psychiatr Serv. 2007;58(10):1330–8. doi: 10.1176/ps.2007.58.10.1330 17914011

[55] Stein BD, Kataoka S, Jaycox LH, Wong M, Fink A, Escudero P, et al. Theoretical basis and program design of a school-based mental health intervention for traumatized immigrant children: A collaborative research partnership. J Behav Health Serv Res. 2002;29(3):318–26. doi: 10.1007/BF02287371 12216375

[56] Elharake JA, Akbar F, Malik AA, Gilliam W, Omer SB. Mental Health Impact of COVID-19 among Children and College Students: A systematic review. Child Psychiatry Hum Dev. 2023;54(3):913–25. doi: 10.1007/s10578-021-01297-1 35013847 PMC8747859

[57] Haight J, Gokiert R, Daniels J. A collaborative, school-based wraparound support intervention for fostering children and youth’s mental health. Frontiers in Education. 2023;8.

[58] Eckert MD, Nishimura ST, Oka L, Barber S, Fleming L, Hishinuma ES, et al. A pilot school-based rural mental health consultation program utilizing an innovative stakeholder partnership at a diverse elementary school. Journal of Rural Mental Health. 2017;41(4):263–83. doi: 10.1037/rmh0000083

[59] Mubarak N, Raja SA, Khan AS, Kanwal S, Saif-ur-Rehman N, Aziz MM, et al. A Conceptual framework of the way forward to a community pharmacist–general practitioner collaborative medication therapy management model for chronic diseases in malaysian primary care: A qualitative study. Risk Management and Healthcare Policy. 2021;14:1615–27. doi: 10.2147/RMHP.S296113 33907479 PMC8064717

